# Power Outages, Extreme Events and Health: a Systematic Review of the Literature from 2011-2012

**DOI:** 10.1371/currents.dis.04eb1dc5e73dd1377e05a10e9edde673

**Published:** 2014-01-02

**Authors:** Chaamala Klinger, Owen Landeg, Virginia Murray

**Affiliations:** Centre for Radiation, Chemical and Environmental Hazards, Public Health England, Chilton, UK; Extreme Events and Health Protection, Public Health England, London, UK; Extreme Events and Health Protection, Public Health England, London, UK

## Abstract

Background Extreme events (e.g. flooding) threaten critical infrastructure including power supplies. Many interlinked systems in the modern world depend on a reliable power supply to function effectively. The health sector is no exception, but the impact of power outages on health is poorly understood. Greater understanding is essential so that adverse health impacts can be prevented and/or mitigated. Methods We searched Medline, CINAHL and Scopus for papers about the health impacts of power outages during extreme events published in 2011-2012. A thematic analysis was undertaken on the extracted information. The Public Health England Extreme Events Bulletins between 01/01/2013 - 31/03/2013 were used to identify extreme events that led to power outages during this three-month period. Results We identified 20 relevant articles. Power outages were found to impact health at many levels within diverse settings. Recurrent themes included the difficulties of accessing healthcare, maintaining frontline services and the challenges of community healthcare. We identified 52 power outages in 19 countries that were the direct consequence of extreme events during the first three months of 2013. Conclusions To our knowledge, this is the first review of the health impacts of power outages. We found the current evidence and knowledge base to be poor. With scientific consensus predicting an increase in the frequency and magnitude of extreme events due to climate change, the gaps in knowledge need to be addressed in order to mitigate the impact of power outages on global health.

## Introduction

In the first three months of 2013, over one million people were reported to be affected by power outages due to extreme events^1^ . Recent examples include the February 2013 flooding in Macedonia^2^, the 2012 Argentine heat-wave^3^, 2012 Hurricane Sandy and the resultant widespread flooding in the USA^4^ and the Japanese Earthquake in 2011^5^ .

Extreme weather events and other natural hazards are increasing in frequency and impact due to urbanisation^6^, and have been shown to have an adverse effect on critical infrastructure including water and sewage treatment, transportation and power supply.

Society’s growing demand for power has extended to the health sector, which is more and more dependent upon electricity to operate safely. Many medical technologies and modern communications are reliant upon power, whilst complex health care is increasingly being delivered in the community. To date, the impact of power loss on health is a poorly studied area and peer reviewed literature is scarce. However, understanding how health is affected by power loss, especially within the setting of extreme events, is vital to future planning so that the health impacts can be prevented and/or mitigated.

The aim of this paper is to identify and describe the health impacts of power outages during extreme events, with the intention of identifying gaps in knowledge and shaping future research strategies. A systematic literature review was carried out, coupled with analysis of the Public Health England Extreme Event Bulletins, to ascertain the magnitude and frequency of power outages on a global scale. Using these approaches, this review details the health impacts of power outages during extreme events and identifies key areas for further work.

## Methods

The content of the published peer reviewed literature from 2011 to 2012 was identified and analysed in order to build material for an initial assessment. To be included in the review, papers had to discuss or describe the health impacts of power outages during extreme events. A key word search in Medline, CINAHL and Scopus was undertaken using the search terms outlined in Table 1.

**Table 1: Search terms used when searching the databases:** 

	Search terms
Extreme event	weather OR "extreme events" OR hurricane* landslide* OR flood* OR drought* OR "heat wave*" OR "ice storm*" OR storm* OR volcan* OR "wild fire*" OR earthquake* OR tsunami* OR "natural disaster*" OR cyclone* OR typhoon* OR avalanche
Power outage	“power outage*” OR “power cut*” OR blackout* OR “ loss of power” OR “ loss of electricity” OR electricity OR power
Health	“health impact*” OR illness* OR mortality OR morbidity OR “public health” OR death* OR disease* OR injur* OR health

Two authors selected relevant abstracts and one author extracted information on the health impacts of power outages and grouped the information into the broad themes of hospital impacts, healthcare impacts, community health impacts and impact on public health infrastructure. Additional articles which fulfilled the inclusion criteria were identified during the search process and by reviewing reference lists. The selected articles were assessed for quality using the Scottish Intercollegiate Guidelines Network (SIGN) guidance^7^. This set of guidelines is used for assessing the quality of evidence during the development of systematic reviews and guidance in public health. It is highly unlikely that research on extreme events and disasters will be the highest level of evidence (i.e. randomised controlled trials and systematic reviews) due to their nature and limited number of peer reviewed reports. However, the SIGN guidelines allows for and recognises this paucity of information. Table 2 describes the levels of evidence described within the SIGN guidance^7^.

**Table 2: SIGN levels of evidence for articles included in literature reviews (Scottish Intercollegiate Guidelines Network, 2011):** 

Level of evidence	Description
1++	High quality meta-analyses, systematic reviews of RCTs, or RCTs with a very low risk of bias
1+	Well conducted meta-analyses, systematic reviews, or RCTs with a low risk of bias
1-	Meta-analyses, systematic reviews of RCTs with a high risk of bias
2++	High quality systematic reviews of case control or cohort studies. High quality case control or cohort studies with a very low risk of confounding or bias and a high probability that the relationship is causal
2+	Well conducted case control or cohort studies with a low risk of confounding or bias and a moderate probability that the relationship is causal
2-	Case control or cohort studies with a high risk of confounding or bias and a significant risk that the relationship is not causal
3	Non-analytic studies, e.g. case reports, case series
4	Expert opinion

Recognising the paucity of the data collected by the systematic review it was decided to undertake a further search to find more evidence on the frequency of reported extreme event power outages. Therefore we analysed the Extreme Events Bulletin^1^ produced by Extreme Events and Health Protection within Public Health England (PHE) (formally Health Protection Agency). News articles are found using a 24 hour Google news search using the following key words: quake, drought, flood, bush fire, smog, hurricane, avalanche, tornado, wildfire, natural disaster, cyclone, mudslide, tsunami, volcano, blizzard, typhoon, landslide, heat wave, famine, snow storm, tropical storm, monsoon, forest fire, storm, wind storm. The bulletins of 1 January – 31 March 2013 were used to identify the frequency, impact, and geographical distribution of power outages. The same time period as the literature review could not be used as the Extreme Events Bulletins for this time period were found to be incomplete.

## Results

**Review of Extreme Events Bulletins: reports of extreme events associated power outages during the first quarter of 2013.**

Fifty two power outages were identified across nineteen countries in this study, all of which occurred as a direct result of extreme events. Table 3 summarises the events identified and the kinds of extreme events which caused power outages. Where reported, the number of people affected by the power outage is also presented.

**Table 3: Occurrence of power outages in extreme events during the first quarter of 2013:** 

Country	Total number of power outages recorded	Extreme Event that caused the power outage(s)	Date	Where available estimated number of people affected
Australia	8	Storm Wildfire Storm Tropical cyclone Bushfire Cyclone Heatwave Tornado	02.01.2013 08.01.2013 16.01.2013 22.01.2013 14.02.2013 25.02.2013 13.03.2013 22.03.2013	- - ~1000 people - ~400+properties - ~1,513 outages -
Bangladesh	1	Tornado	24.03.2013	-
Brazil	1	Drought	06.01.2013	-
Bulgaria	1	Strong winds	25.03.2013	-
Canada	5	Strong winds Strong winds Heavy snow, high winds Snow High winds	20.01.2013 01.02.2013 18.02.2013 28.02.2013 20.03.2013	~26,000 people 100,000 customers ~1,000 customers 30,000+ homes 32,000+ people
Chile	1	Earthquake	31.01.2013	-
China	2	Earthquake Tornado	04.03.2013 20.03.2013	- -
France^*^	3	Cyclone Cyclone Winter storm	03.01.2013 03.01.2013 12.03.2013	60,000+ homes ~3,200 people 68,000+ homes
Greece	1	Torrential rain	22.02.2013	-
Hungary	1	Snowstorm	15.03.2013	100,000+ people
India	3	Freezing cold conditions Heavy rains/storm Cold wave	04.01.2013 07.02.2013 26.03.2013	- - -
Indonesia	1	Tropical cyclone	28.02.2013	1,000+ people
Israel, Palestine & Jordan	1	Snowfall	10.01.2013	1,000+ homes
Macedonia	1	Flooding	27.02.2013	~20,000 people
New Zealand	2	Tornado-rain-floods Strong winds	10.01.2013 17.03.2013	- -
Pakistan	3	Heavy rain Torrential rain Rain	27.02.2013 28.02.2013 28.03.2013	- - -
Soloman Is.	1	Earthquake & Storm surge	06.02.2013	-
UK	2	Strong winds Heavy snow	31.01.2013 22.03.2013	- 211,300+ homes
USA	14	Snow Winter weather Strong winds Storm Severe Storm Snowstorm Tornado Hurricane force blizzard Snowstorm High winds High winds Snow Dust storm/Tornado Hurricane force winds	18.01.2013 18.01.2013 20.01.2013 28.01.2013 30.01.2013 11.02.2013 11.02.2013 25.02.2013 27.02.2013 27.02.2013 05.03.2013 06.03.2013 17.03.2013 24.03.2013	2,300 customers - 2000+ residents ~1,125 customers 60,000+ customers 113,844+ properties - 10,000 people 50,000+ homes ~902 customers 10,000+ customers ~250,000+ people - -
				* including overseas territories

The mixed nature of extreme events causing power outages is illustrated in Figure 1. Although storms, winds and snow account for the majority of power outages, many other types of extreme events for example flooding were also as responsible for such outages but to a lesser extent.

**Power outages by country and hazard classification (1st January to 31st March 2013):**
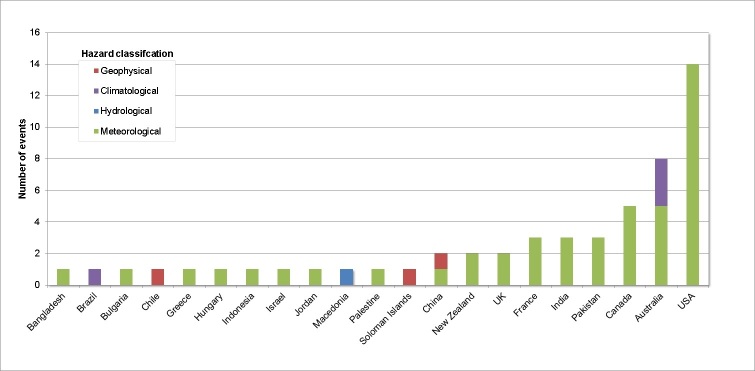

**The literature review: health impacts of power outages during extreme events**

Of the initial 125 papers identified through the keyword search, 8 duplicates were removed and 91 papers were excluded from the investigation because they were found to be not relevant from closer scrutiny of the title and abstract. A further 9 articles were excluded on reading of the full text articles as they were found to not relate to the health impacts of power outages, leaving 17 articles which fulfilled the inclusion criteria. An additional 3 papers were discovered through the reviewing of references and during the search process.

Figure 2 summarises the flow of articles during the search process as per the PRISMA guidelines^8^.

**PRISMA flow diagram:**
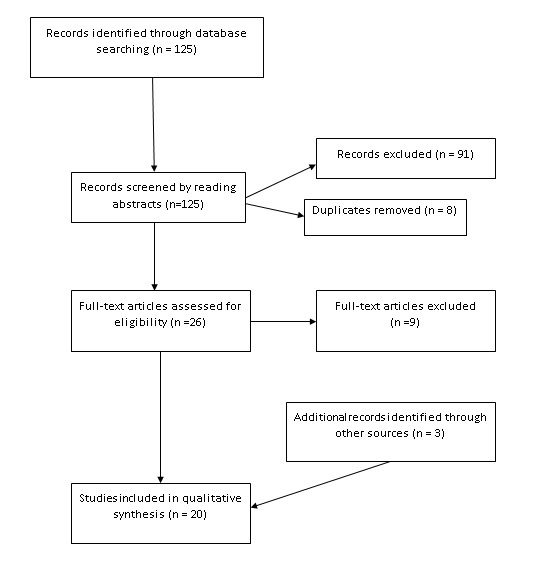

The evidence level for the reviewed articles, according to SIGN guidelines, is summarised in Table 4 below.

**Table 4: the level of evidence for the articles included in the review:** 

References	Evidence Level	Description
9 ^,^ 10 ^,^ 11 ^,^ 12 ^,^ 13 ^,^ 14 ^,^ 15 ^,^ 16 ^,^ 17 ^,^ 18 ^,^ 19 ^,^ 20	4	Mainly descriptive accounts of power outages occurring during extreme events and how they impacted on health and healthcare
21 ^,^ 22 ^,^ 23 ^,^ 24 ^,^ 25 ^,^ 26	3	Some analytical research, mostly regarding the prevalence of carbon monoxide poisoning during power outages
24 ^,^ 27 ^,^ 28	3	Some analysis of emergency data and survey data regarding food safety during power outages
29	1+	A systematic review of the literature regarding carbon monoxide poisoning during disasters

As can be seen by Table 4 above, the evidence base in this field is poor, with most of the literature being expert opinion. In many instances, the articles had to be carefully scrutinised to extract information relating to health impacts. Sometimes the mention of the health impacts was vague and not extensively elaborated. For example, one study states that ‘critical care devices…. were malfunctioning’^13^, but does not elaborate on which types of devices were problematic. The issue of power outages leading to the loss of light was mentioned by some authors^10^^,^ 13 ^,^ 16, but they did not elaborate the impact darkness had on patient care.

Another study explains that when the Christchurch earthquake occurred, the Christchurch Intensive Care Unit (ICU) was staffed to take 15 ventilated patients^16^. The 14 patients who were already in ICU were transferred out during the first 24 hours, only to be replaced by 18 earthquake related patients. However, the author does not go into details as to how many of these patients required ventilation and what special challenges were faced during the power outage^16^. Two studies note that ventilated patients were evacuated from flooded hospitals during Hurricane Sandy^12^^,^ 14 and one mentions that ‘oxygen tanks and interns were stationed at the bedside of ventilated patients’^14^.

Many health impacts (for example loss of transport and communication) were evident across different settings and a complex web of interactions was revealed in the literature. Health impacts which frequently recurred across different settings are summarised in Figure 3.

**What do we lose when we lose power?:**
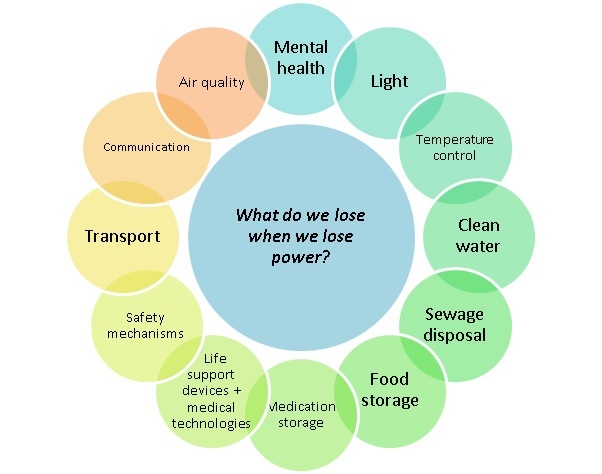

Table 5 categorises the health impacts of power outages in different settings.

**Table 5: A typology of health impacts of power outages:** 

Category	Impact
**Hospital**	**Direct clinical care:** Malfunctioning critical care devices led to decision to evacuate a hospital^13^ Patients on ventilators had to be evacuated^12^^,^ 14 ^,^ 16 A Ventricular Assist Device was not functioning and ran on battery power and a backup generator ‘nursed by the National Guard.’ The heart surgery team stayed with the patient until he could be moved to a hospital with power^12^ Intra-venous drips were converted to subcutaneous drips where possible to save on electricity^14^ Lifts were not working and patients had to be evacuated down the stairs^10^^,^ 12 ^,^ 14 ********
	**Patient records and identification:** Accessing clinical records was difficult^10^^,^ 17 e.g. patients’ viral status^10^ Generating patient identifications was compromised, leading to difficulties tracking patients and patient results^16^
	**Increased demand on services:** Emergency Department (ED) was short staffed whilst volumes of patients doubled^12^ Patients on home oxygen had to seek oxygen at hospital as their oxygen concentrators were not working^9^^,^ 19 Influx of patients needing electricity to recharge equipment 9 respiratory treatments^9^ dialysis. For example, 27 patients were waiting for dialysis in one ED at one point during Super Storm Sandy^10^^,^ 12 insulin^19^ Elderly patients with no access to home nursing**10**
	**Communication systems Patients communicating with hospital:** Patients unable to dial 911 to get emergency help due to loss of telephony (landlines and mobiles)^10^ **Within the hospital:** Runners were used to relay messages^14^ Inability to charge phones^17^ Doctors downloaded and used a telephony app as the wi-fi system continued to not function^10^ **Hospitals communicating with the outside world:** Communications systems were ‘overloaded and not working’ – therefore staff were unable to judge the extent of the event and prepare^16^. Once power was restored, medical students were sent to watch TV news and bring back updates on the situation. Inability to charge phone^17^ Staff spelled “SOS” with bed sheets on a hospital roof in an effort to get outside help^17^
	**Hospital infrastructure dependent on electricity:** Heating, cooling, gas and air handling, washing linen and dishes, cooking. For example, one hospital used disposable utensils and plates, reduced the changing of bed linen and changed the menus to reduce power usage^17^ Blood bank had no power^16^ Radiology department had no power^16^
	**Transport to and from the hospital:** No public transport (no buses, subways) for patients and staff to use**10^,^^12^**
**Healthcare**	Loss of home oxygen supply 9 ^,^ 10 ^,^ 15 ^,^ 19
	Nebulisations failed leading to ‘asthma exacerbations and shortness of breath’^10^
	Loss of functioning ventilators^9^^,^ 10
	Dialysis sessions missed leading to ‘life threatening hyperkalaemia’^10^
	Drug storage compromised^9^^,^ 10 ****. For example: Biological therapy for rheumatic conditions^20^ Insulin^19^ Vaccine**11**
	Lifts and Hoists not working leading to difficulties in caring for vulnerable patients in the community^10^^,^ 15
	**Transport:** Patients getting to hospital^10^^,^ 12 Patients calling emergency services for help^10^ Staff accessing patients in the community^10^^,^ 15
	**Communication:** Mobile phone service and landlines not working^10^^,^ 12
**Community health effects**	Carbon monoxide poisoning caused by the unsafe use of generators for electricity, and grills and gas-powered heaters for cooking and heat generation^10^^,^ 21 ^,^ 22 ^,^ 23 ^,^ 24 ^,^ 25 ^,^ 29
	Electrocution^19^
	Adverse mental health- loss of services (including electricity) was significantly related to depression, anxiety and Post Traumatic Stress Disorder^26^
**Loss of public health infrastructure**	**Clean water:** Loss of water monitoring and pumping mechanisms^19^ Water boil notices and water restrictions were issued^18^
	**Sewage treatment:** Sewage treatment failure could lead to untreated/ undertreated sewage being added directly to streams^19^ Water treatment is reliant on power^18^
	Food storage and safety^17^^,^ 18 Meal providers had to provide shelf stored food as fridges didn’t work^19^
	Loss of safety mechanisms – national parks were shut due to loss of fire suppression systems. Traffic lights were not working^19^
	**Temperature control:** Inability to ‘get relief from the heat’^18^ Lack of heating is a problem for “elderly, homebound patients and small children, especially in low income housing projects”^13^ People tried to ‘heat their homes with kitchen stoves’^10^

## Discussion

Despite power outages being a significant public health issue with a considerable impact on health, to date very little has been published regarding this issue in the peer reviewed literature. It is apparent that there is now increasing awareness and interest in this issue. The extreme events bulletin has been a recent and invaluable resource, collating available data in an accessible format.

The following sections discuss the key findings of the review. A précis of the limitations of this review is presented, followed by gaps in research and some practical solutions on how to respond to power outages.

## Key findings

**Frequency of power outages associated with extreme events**

There were fifty two reports of power outages across nineteen countries caused by extreme events during the first three months of 2013. Overall, the results from the bulletins search gave an impression of how frequent and widespread power outages are during extreme events. The events and outages occurred in both economically developed and less developed nations and in island and mainland state settings. It is interesting to note that none of the media reports contained any reference to health impacts.

The surprisingly high number of power outages reported in the PHE Extreme Events Bulletin is likely to be an under-representation of worldwide power outages. This is despite the news articles included in the PHE Extreme Events Bulletin being collected from several international media sources. These media sources include Google News, BBC, UK broadsheets and international webpages including PreventionWeb, UNISDR and ReliefWeb. While this ensures an international perspective, it also introduces a reporting bias towards more economically developed countries. This may account for the fourteen reports of power outages in the USA alone, whilst there were no reports from Africa. The media search was only conducted over a three month period. Seasonal variations in power outages can only be found with a longer search period. However, as our search was not limited to one hemisphere, different seasons have been represented to some extent.

**Health care**

The impact of power outages on health is varied and far reaching. From the first call for help to the giving of complex clinical treatments, it is evident that healthcare is increasingly dependent on power. Electricity was recognised by the UK Department of Health as the ‘most vital of all infrastructure services’ because ‘without it most other services will not function’^30^. A survey conducted in Japan found that 65% of disaster base hospitals (i.e. hospitals which are responsible for supporting other hospitals during a disaster) considered electricity to be the most vital lifeline for the functioning their hospital. This survey also revealed that 60% of these hospitals felt that key services such as emergency surgery and heamodialysis would have to be stopped if generator power was unavailable. Key equipment related services such as laboratory services, imaging and sterilisation would also be stopped if generators failed^31^. Most hospitals have generator backup for only eight hours. However, in longer term power outages, hospitals can be faced with limited fuel and difficulties in sourcing fuel for generators, due to transportation and communication difficulties.

The literature suggests that during an extreme event, not only do hospitals have to deal with the usual intake of patients, but also with disaster related patients and patients with chronic illnesses who do not have access to the medical technologies they need^12^^,^ 13 ^,^ 16. There is an increasing trend for people with functional needs (for example, people with respiratory illnesses needing oxygen or nebulisers) to be cared for in their own communities in the USA^9^; a trend echoed in the UK and across the world^32^^,^ 33.

In an era of increasing digitalisation of patient records, accessing electronic patient records and generating patient identifiers can be problematic without power^16^. However, in some instances using a distant server or cloud computing as a backup has meant that patients can be cared for at different locations with good access to their records. One study describes how patients continued to receive prescriptions and scheduled treatments as planned during Hurricane Sandy^9^, while another states that electronic medical records were ‘vital’ for continuing the rheumatology service after the 2011 Christchurch earthquake^20^.

**Basic public health infrastructure**

The complexities of interactions between power and other aspects of society cannot be underestimated. One study recounts how the 2003 New York blackout affected water supply as the water pumps were reliant on power^34^. The public had to be informed that they should use boiled or bottled water. But the mechanisms for communicating to the city were also not functioning because of the blackout and even if the message were communicated, people had no means of boiling water^34^. This example illustrates how the lack of power can have a domino effect and impact on health at many levels. This was also the case in hospitals affected by Hurricane Rita (2005), where fuel shortage led to power outages and consequently loss of power led to reduced water pressure^35^.

**Light**

Some authors noted that the loss of electricity also meant the loss of light^10^^,^ 13 ^,^ 16. Light is essential to the provision of safe and effective healthcare. From basic clinical observations to more complex interventions such as operations, clinicians rely on a good light source. Although the impact of the loss of light on health was not directly documented in the literature, the importance of light in everyday clinical care is obvious. In a survey of 213 hospitals affected by the 2011 Japanese tsunami, 121 hospitals had temporary loss of power, whilst in 12 hospitals the emergency power generators did not work at all. This study demonstrates the impact of power outages and the increased risk of operations being discontinued (odds ratio 110.52, 95% confidence interval 8.91 – 1371.12, p < 0.001)^36^.

Even in the community setting, light prevents accidents in the home and provides a measure of comfort and reassurance to people who are otherwise alone and vulnerable.

**Carbon monoxide poisoning**

Incorrect use of generators during extreme events has led to peaks in carbon monoxide poisoning^21^^,^ 22 ^,^ 23 ^,^ 24 ^,^ 25 ^,^ 29. One study reports that the most common cause of disaster related carbon monoxide poisoning was generator use accounting for 54% of non-fatal cases and 83% of fatal cases^29^. Other causes of carbon monoxide poisoning during disasters included heaters, stoves and grills^29^.

**Food safety**

Power outages leading to an increase in diarrhoeal diseases has been documented in the past^37^. One study focused on food safety during power outages and found that US citizens are poorly prepared^27^ . This poses the question of how well people are prepared for power outages in resource poor settings. On the one hand, backup mechanisms may be poor or non-existent. However, it may be that resource poor settings are less affected as they are less reliant on electricity than their resource rich counterparts. For example, whilst it is common practice for people in high resource countries to eat refrigerated food, this is probably an uncommon way of storing food in resource poor settings. From this point of view, there must be lessons to be learnt from resource poor settings on how best to adapt to life without power and this is an area which should be explored.

**Other identified issues**

Several issues were not discussed in the peer reviewed literature but became apparent through discussions with colleagues and reading of the wider grey literature.

Power outages can lead to social isolation especially in vulnerable groups. For example, there were accounts of elderly people being isolated in high rise flats in New York during Hurricane Sandy, as the lifts were not working^38^.

The confusion and agitation felt by patients with dementia when faced with sudden, unexplained darkness can be imagined, though not documented in the literature.

Most hospital generators are set to provide back up power for only 8 hours, but major power outages frequently last much longer. Hospital generators are also set up to provide power for key services and needs. For example, power for the computer network would be prioritised over power to air conditioning. However, loss of air conditioning can lead to excessive heat which in turn leads to computers automatically switching off. This was an issue which was identified by some hospitals during the Great East Japan Earthquake.

Many medicines should be stored at 4- 25 ⁰C, which can be problematic in the community and in healthcare settings, if power outages occur during heat waves.

## How can we prepare for power outages?

An overall approach to resilient hospital power supplies was suggested by the UK Department of Health, with the use of standby generators being just one aspect of resilience^30^. It was suggested that electrical distribution systems need to be robust enough to survive threats and hazards and that single points of failure should be eliminated where possible. Other suggestions include having more than one regional electrical provider, the ability to reconfigure failed supply systems and a set of actions and automated responses which are triggered in the events of mains supply failure^30^. The location of hospitals requires careful consideration, to ensure that hospitals are built within low hazard (for example low risk of flooding) so that disruption of infrastructure, including power, is minimised or prevented. Similar recommendations for maintaining a resilient power supply are made by Pourbeik et al, in their analysis of the 2003 US-Canadian blackout which left 50 million people without power. The authors identify failure of aging equipment, lack of reliable real time data and lack of automated controls to prevent cascading of events as root causes, recommending regular maintenance of equipment, prioratising replacement of old equipment and the use of new and emerging technologies to mitigate the effects of such an event.^49^

Some authors have made suggestions on how proper planning can mitigate the effects of power outages on health. One study states that shelters turned away patients who simply needed oxygen or the ability to recharge the batteries of their medical equipment because the shelters were poorly prepared for these simple medical needs^9^. Such patients would then flood into emergency departments which have already been put under strain^9^. In contrast, during the 2008 Hurricane Ike in Ohio, the Red Cross provided information via the media on where patients could obtain electricity for oxygen converters^19^. Shelters need to be equipped to charge up batteries or give oxygen so that patients who normally manage their illnesses at home can be managed in a shelter and do not necessarily have to seek hospital care. Communicating these messages during an extreme event is problematic^34^, so public awareness needs to be enhanced before such events occur.

Another study suggests that power companies should have a list of patients using medical technologies in the community, so that their power needs can be prioritised in the event of an emergency^9^. In the UK, energy companies are obliged to maintain Priority Service Registers for vulnerable populations including people reliant on medical technologies^43^^,^ 39. However, patients need to be aware of the register to be able to request registration. For example, in a survey of patients requiring dialysis, only 38% of patients requiring home peritoneal dialysis had notified the local electricity supplier of their health condition^44^.

Awareness of alternative methods of managing chronic diseases during emergencies needs to be increased amongst patients and healthcare providers. For example, patients requiring dialysis can increase the inter-dialysis interval by maintaining a special diet which limits protein, potassium, sodium and fluid intake^44^. However, only 57% of dialysis patients were aware of this emergency diet during an American survey undertaken by Foster et al^44^. Despite disaster related rises in carbon monoxide poisoning being documented in the literature, there were nine deaths caused by carbon monoxide poisoning during Hurricane Sandy^41^. This highlights the importance of public education prior to, during, and after an extreme event. Similarly, public education on food safety and what to do with refrigerated and frozen food will prevent an increase in diarrhoeal illness.

Within secondary care, although many hospitals will have backup generators which supply power in the event of an emergency, this is not in itself a panacea. Some generators have stopped working during extreme events because they were situated in flooded basements^40^ whilst others were situated higher up but the fuel pumps got flooded requiring ‘bucket brigades’ to carry 5 gallon jugs of fuel up to a fuel tank on the 13^th^ floor^12^^,^ 13 ^,^ 14. This was reiterated by another study after an investigation of hospital preparedness and response during Hurricane Rita^35^ . Therefore, hospital emergency planners need to be meticulous in their planning.

One study suggests that hospitals must plan for longer term power losses, when washing, cleaning, communications and cooking could also be affected, as power is redirected to clinical areas^17^. Another study found that five hospitals lost power for a mean of 4.8 days (range 0.5 to 11 days) during Hurricane Rita^35^; again highlighting the importance of planning for longer term power outages.

One study advises that policies to decrease dependence on traditional sources of power are needed and the experience and ingenuity of those living and working in resource poor settings could be invaluable^9^. This study also suggests that policy makers need to consult patients with chronic diseases about emergency preparedness and planning so that their expertise and insights can be incorporated within the decision-making process^9^.

## Limitations

The literature search revealed a dearth of high quality research in this field, with most of the published articles being evidence level 3 and 4. For resource reasons the literature search was limited to 2011 and 2012. Although the language was not limited to English, only one non-English article was found which did not meet the inclusion criteria. This is probably the first review of this important aspect of extreme events preparedness and it provides a much needed initial assessment. Reviewing literature from a longer time period may reveal other health impacts, but may also contradict some of the present findings or reduce the prominence of some impacts identified here.

Publication bias in the peer reviewed literature, the grey literature and the media needs to be considered. There may be a preponderance of reporting of extreme events and power outages which occur in higher income countries. For example, although there were published articles on extreme events occurring in the USA, the floods which occurred in Thailand in 2011 have not featured in the peer reviewed literature yet. However, reports of power outages and deaths from electrocution during these floods have been presented at a conference^48^.

Inconsistency in terminology when describing extreme events and health impacts is another limitation. As already described in the results section, in many instances, health impacts were buried deep within the peer reviewed papers and in the case of the media, the health impacts were not mentioned at all.

## Gaps in research

Although there are descriptions of power outages in extreme events and the health impacts can be gleaned from these accounts, there are very few attempts to quantify health impacts in terms of morbidity, mortality or quality of life. The only area where quantification has been attempted is in the field of carbon monoxide poisoning during extreme events. Although research during extreme events is difficult, it needs to be done so that we can learn from these events and improve resilience for the future.

As with any public health issue, a key question is how to influence and change people’s behaviour, especially in extreme situations where people are faced with unusual stressors. Further research is needed on the most effective methods of communicating risks and safety messages during extreme events, so that population behaviour is influenced to improve health.

Scientific innovation in medical technologies and treatments is needed, so that those with functional needs are more resilient to extreme events. Examples include the development of medical technologies that can run on a sustainable power source, such as solar power, and medications which can be stored at all ambient temperatures.

Finally, there is a wealth of experience within the global health community of facing extreme events and solutions which have been successful in overcoming power outages. This collective knowledge and experience is a valuable resource which must be explored so that innovative and effective strategies can be shared and utilised.

## Conclusions

As far as can be determined, this is the first review of the health impacts of power outages occurring during extreme events. This review has demonstrated that this area has a poor evidence base and that research into this important issue is scarce. In the light of increasing demand and reliance, power outages will continue to have far-reaching impacts upon the health of vulnerable populations who are increasingly reliant on electrically powered technology. Whilst ascertaining the morbidity and mortality which can be directly attributed to power outages associated with extreme events is challenging, the results of this review illustrate the difficulties faced when attempting to provide a safe healthcare provision during extreme events. Emergency Preparedness Professionals must plan for extended power outages and the healthcare workforce must be aware of what resources are available during such emergencies. Innovative medical technologies and treatments are needed, so that those with functional needs are more resilient to extreme events. Lessons must be learnt from resource poor settings where healthcare is regularly provided despite power shortages.

With scientific consensus predicting an increase in the frequency and magnitude of extreme events due to climate change, the gaps in knowledge identified within this review need to be addressed in order to mitigate the impact of power outages upon global health.

## Competing Interests

Virginia Murray is the head of Extreme Events and Health Protection which would make use of this data in their daily work. The authors declare that no other competing interests exist.

## Appendix 1

### PRISMA Checklist

Download PDF
